# Supplementary material for: 5-HT1A Receptor Agonist Treatment Partially Ameliorates Rett Syndrome Phenotypes in mecp2-Null Mice by Rescuing Impairment of Neuron Transmission and the CREB/BDNF Signaling Pathway
Source: Int J Mol Sci. 2022 Nov 14;23(22):14025. doi: 10.3390/ijms232214025 (PMC9697184; doi:10.3390/ijms232214025)
Supplement: Supplementary file 1 [file ijms-23-14025-s001.zip › ijms-1997542-supplementary.pdf]

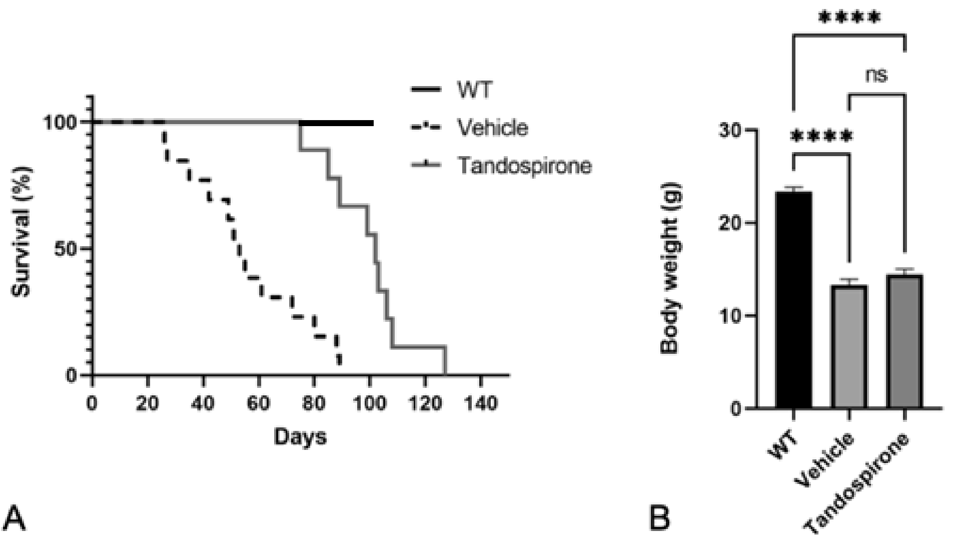

Supplementary Figure S1. Effect of tandospirone treatment on the *Mecp2*-KO phenotype. Both *Mecp2* WT and *Mecp2*-KO (Vehicle) mice were treated with saline. (**A**) Lifespan was measured using Kaplan-Meier survival curves, which show the proportion of mice that survived (y-axis) at each day after birth (x-axis) for *Mecp2*-KO saline-treated (Vehicle) and *Mecp2*-KO tandospirone-treated (Tandospirone) mice. *Mecp2*-KO mice (Vehicle) gradually died between P28 and P84 days, but the tandospirone treatment group exhibited a significantly longer lifespan than the vehicle group ($p < 0.05$; vehicle: n=30, tandospirone: n=12). (**B**) Body weights were measured at 8 weeks of age (n=12~15). No effect on body weight was observed after tandospirone treatment. **** $p < 0.0001$, ns: not significant.

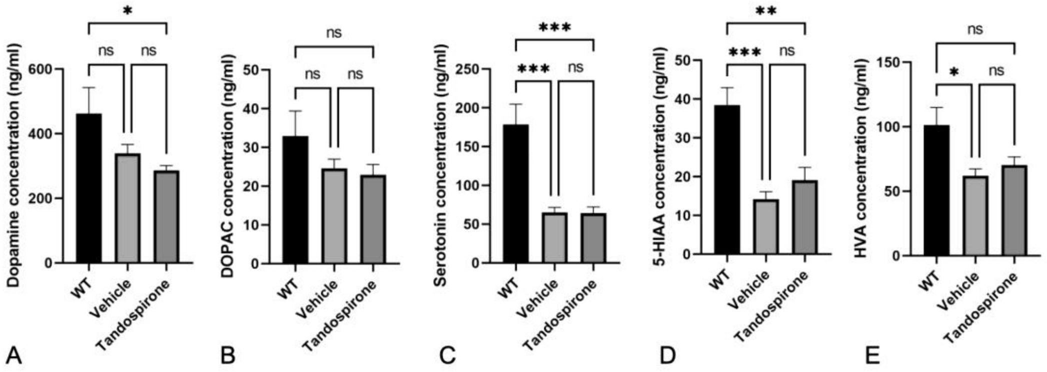

Supplementary Figure S2. Effect of tandospirone treatment on dopamine, serotonin and their metabolites in the cortex. *Mecp2*-KO mice showed lower dopamine (**A**) and DOPAC (**B**) concentrations than WT mice, but the difference was not significantly in the cortex. The contents of serotonin (**C**), 5-HIAA (**D**) and HVA (**E**) were significantly decreased in the *Mecp2*-KO mice compared with the WT mice. Tandspirone had no effect on the dopaminergic or serotoninergic systems in the cortex. All values are expressed as the means ± SEMs. Statistical analysis was performed using one-way analysis of variance (ANOVA) with Tukey's multiple comparison post hoc test for intergroup comparisons. n=6, * $p < 0.05$, ** $p < 0.01$, *** $p < 0.001$, ns: not significant.

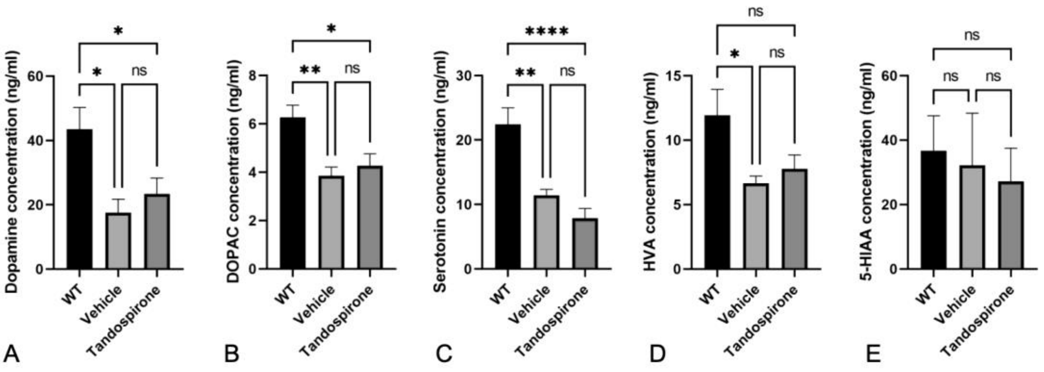

Supplementary Figure S3. Effect of tandospirone treatment on dopamine, serotonin and their metabolites in the midbrain. *Mecp2*-KO mice showed significantly lower dopamine (**A**) and DOPAC (**B**) concentrations than WT mice in the midbrain. The contents of serotonin (**C**) and HVA (**D**) were significantly decreased in the *Mecp2*-KO mice, but there are no changes in the 5-HIAA (**E**). There was also no effect of tandospirone on the dopaminergic or serotoninergic systems in the midbrain. All values are expressed as the means ± SEMs. Statistical analysis was performed using one-way analysis of variance (ANOVA) with Tukey's multiple comparison post hoc test for intergroup comparisons. n=6, * $p < 0.05$, ** $p < 0.01$, **** $p < 0.0001$, ns: not significant.

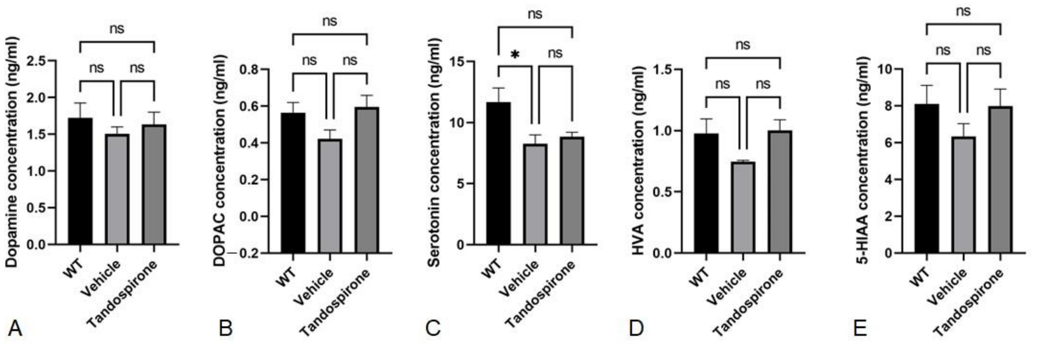

Supplementary Figure S4. Effect of tandospirone treatment on dopamine, serotonin and their metabolites in the hippocampus. *Mecp2*-KO mice showed lower dopamine (**A**), DOPAC (**B**), serotonin (**C**), HVA (**D**) and 5-HIAA (**E**) concentrations than WT mice in the hippocampus, but only serotonin concentrations are significantly lower. There was also no effect of tandospirone on the dopaminergic or serotoninergic systems in the hippocampus. All values are expressed as the means ± SEMs. Statistical analysis was performed using one-way analysis of variance (ANOVA) with Tukey's multiple comparison post hoc test for intergroup comparisons. n=6, * $p < 0.05$, ns: not significant.

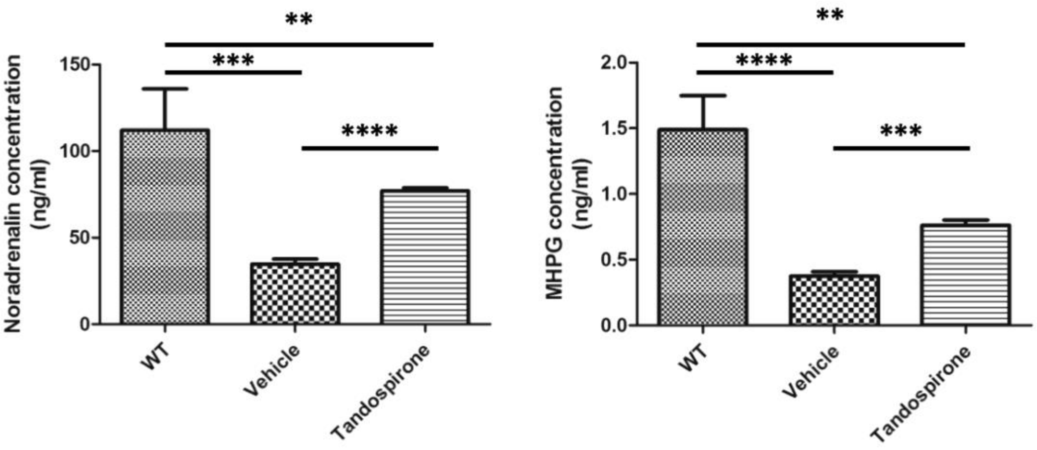

Supplementary Figure S5. Noradrenalin and its metabolite (MHPG) concentrations in the brainstem. The concentrations of noradrenalin (A) and MHPG are recovered with tandspirone in the *Mecp2*-KO mice. WT; wild-type mice, Vehicle; *Mecp2*-KO mice, Tandspirone; tandspiron-treated *Mecp2*-KO mice. n=6, ** $p < 0.01$, *** $p < 0.001$, **** $p < 0.0001$

Supplementary Table S1. Primer list for quantitative PCR.

**Gene**	**Primer sequence**
Camk2d	Forward: 5′-GGTGGCGAACTGTTTGAAGA-3′
	Reverse: 5′-GATGGACTACGCCCATCTGA-3′
Prkag2	Forward: 5′-ACCTTCAAGCCTTTGGTGAAC-3′
	Reverse: 5′-AGTGCGTTCCCACTGATAGG-3′
Creb1	Forward: 5′-AACCAGCAGAGTGGAGATGC-3′
	Reverse: 5′-GCAGATGATGTTGCATGAGC-3′
Bdnf	Forward: 5′-GAGCCGAACAAACTGATTGC-3′
	Reverse: 5′-GCCTTCATGCAACCGAAGTA-3′
Mapkapk2	Forward: 5′-TCGACAAGAGAACCCAGCA-3′
	Reverse: 5′-AGTGCAGCTCCACCTCTCTG-3′
Gabra4	Forward: 5′-CTATGGATGGTCATGCCTGC-3′
	Reverse: 5′-AAGACTCCTTTGGCACCTCC-3′
